# New Muscle in the Eye

**Published:** 1847-09

**Authors:** 


*New Muscle in the Eye.*—The *Oest. Med. Woch.*, No. 40, mentions that in the human eye and also in that of the mammifera, Dr. Brüche states that he has discovered a new muscle. This muscle appears in a manner to have the same power as that of the iris. It consists in the grey ring which is found in front of, and in the neighbourhood of the iris on the external surface of the choroid, and which has been usually known under the name of the ciliary circle. Brüche gives to this muscle the name of the expansor muscle of the choroid.—*Monthly Journal of Medical Science, from Gaz. Med. di Milano.*

